# Case report: Cheek acupuncture exhibits an immediate effect in relieving severe pain associated with nerve compression or damage of central nervous system and its potential mechanism of action

**DOI:** 10.3389/fnins.2023.1211361

**Published:** 2023-07-20

**Authors:** Yongzhou Wang, Lu Yang, Yongzheng Wu

**Affiliations:** ^1^International Cheek Acupuncture Therapy Institute, Fontenay-sous-Bois, France; ^2^Department of Anesthesiology, Beijing United Family Hospital, Beijing, China; ^3^Unité de Biologie Cellulaire & Infection Microbienne, CNRS UMR3691, Institut Pasteur, Université Paris Cité, Paris, France

**Keywords:** pain, central nerve system, nerve compression, cheek acupuncture, mechanism of action

## Abstract

Peripheral nerve compression or permanent damage of central nervous system (CNS) can trigger severe neuralgia to patients. Analgesic medicine or even surgery to remove nerve compression is commonly used for pain relief. But these treatments either are ineffective, have side-effect or can cause subsequent complications. Acupuncture, a technique that has been widely used in China and other Asian countries for thousands of years, is an alternative to relieve pain, although the mechanism of action is not fully understood. In this study, two patients who had symptoms of severe neuralgia associated with peripheral nerve compression or permanent damage/dysfunction of CNS and analgesic medicines are ineffective, underwent cheek acupuncture, a new technique established recent years by the author with the features of painless, standardization, simplicity, and precision. An immediate analgesic effect of the cheek acupuncture was observed without any side effects, and clinical remission was achieved after several sessions of treatments. It suggests that this new approach is an efficient alternative for pain relief induced by nerve impairment. The authors proposed a biological holographic model of triplet homunculi existing at the level of the local cheek, spinal cord, and cerebral cortex, to explain the immediate and accurate analgesic effect of the cheek acupuncture. These homunculi have the same structure, and synchronized sensations and actions that are mediated by afferent and efferent neurons, as the integrated human body. Therefore, the nociception and needling signals are sensed, transmitted, analyzed, and manipulated cooperatively and simultaneously among these homunculi with the subsequent pain relief in the body.

## Highlights

Relieving severe pain resulting from peripheral nerve compression or permanent damage/dysfunction of the CNS is a major challenge for public health. Analgesic medicines or even surgery are commonly used for pain relief; however, these methods either are ineffective, have side effects or can cause subsequent complications. Thus, it is essential to pursue an alternative approach to relieve the pain associated with nerve impairment. Acupuncture has been applied for thousands of years in China for pain relief or other diseases. But more than 300 acupoints of traditional acupuncture make it complicated to learn and manipulate. This study reports that cheek acupuncture, a new technique established by the author, has immediate and accurate analgesic effect on the pain associated with a disorder of the nervous system, and is almost painless during needling, when analgesic medicines are ineffective. This technique contains only 16 standard acupoints on each cheek, whose orientation is precisely determined based on the anatomic structure of the skull. Therefore, cheek acupuncture represents an efficient strategy to relieve the pain induced by the impairment of the nervous system. We propose a biological holographic model of triplet homunculi located on the cheek, spinal cord, and CNS to interpret the immediate and accurate analgesia of this technique.

## Introduction

Pain is “an unpleasant sensory and emotional experience associated with, or resembling that associated with, actual or potential tissue damage” (Raja et al., 2020), which is experienced by everyone in their lifetime. Based on the duration or suffering time, pain can be divided into acute and chronic pain. Acute pain can be considered as the normal physical response of the body to a stimulus and is relieved once the body recovers (Ashburn and Staats, 1999; Carr and Goudas, 1999), while chronic pain is defined as pain lasting for more than three to six months (Treede et al., 2019). It is estimated that almost half of the patients pursuing for medical care each year in the USA are due to pain, and that one in four Americans suffers from chronic or recurrent pain (Turk and Melzack, 2011). Similarly, chronic pain is diagnosed in nearly 20% of Europeans based on a survey among 16 countries (Breivik et al., 2006). Pain severely affects the quality of life and the ability to work of patients and can even cause psychological disorders. Therefore, pain and its treatment are major challenges in clinical medicine and public health.

Nerve pain (neuropathic pain or neuralgia) is a particular type of pain with an electric shooting-, stabling- or burning-like sensation. Peripheral nerve compression/pinching or damage of the CNS is one of the common causes of this disease. Various disorders can cause the compression or damage of nerves, including accidents and trauma, infection/inflammation, bone fractures, herniated discs, surgical complications, tumors, cerebral hemorrhage, and ischemic stroke, etc (Bendszus and Stoll, 2005). Nerve compression or pinching can last for several days to a couple of years. But severe and persistent compression of peripheral nerve or CNS damage can eventually lead to severe pain, irreversible muscle loss, and permanent nerve damage with subsequent dysfunction of the tissues or organs.

Despite surgical decompression of compressed nerves or improvement of blood supply in the brain of patient with stroke, prescription and non-prescription analgesics, including non-steroidal anti-inflammatory drugs (NSAIDs), steroids, opioids, paracetamol, COX-2 inhibitors, and more, have been widely used to relieve pain (Breivik et al., 2006). However, long-term use of these medicines can cause side effects such as addiction and resistance. Additionally, the pharmacological strategy is often ineffective, particularly for severe neuralgia, leading to sustained or recurrent pain in many cases (Breivik et al., 2009). For these reasons, more and more patients seek non-medication treatments such as massage and physical therapy to relieve pain. Among these alternatives, acupuncture has been widely used for thousands of years in China and other Asian countries for pain relief and other diseases. In 1979, a symposium on acupuncture organized by the World Health Organization (WHO) recommended 43 diseases suitable for acupuncture therapy (World Health Organization, 1979). Since then, the analgesic effect of this technique has been approved by several national institutes of health (NIH), such as the NIH of the US in 1997 (National Institutes of Health, 1997), and more clinical trials on acupuncture have been conducted worldwide for hundreds of diseases or conditions including pain relief (World Health Organization, 2002).

Microneedling therapy belongs to one of the acupuncture methods, involving the needling of a special organ's local surface or a specific location on the body to treat diseases initially occur in another part of the body. The principle of facial acupuncture, the first microneedling system, was described thousands years ago in “The Yellow Emperor's Classic of Internal Medicine (also referred to as Huangdi Neijing)—Miraculous Pivot: Chapter of Five Colors.” Nowadays, various microneedling systems have been established and widely used in China and other countries, including scalp acupuncture, ear acupuncture, abdominal acupuncture, wrist-ankle acupuncture, and cheek acupuncture, etc (Xu et al., 2019). In particular, cheek acupuncture developed by Wang (2017), has been confirmed to effectively and immediately relieve pain associated with a range of diseases (Wang et al., 2000; Ren et al., 2014; Wang, 2017; He and Li, 2018; Cai et al., 2020; Ding et al., 2020; Sun et al., 2022).

In this study, we report on cheek acupuncture used to alleviate severe neuralgia caused by peripheral nerve compression or permeant damage/dysfunction of CNS in patients with an immediate analgesic effect. The potential mechanism of action of this new technique is also discussed.

## Case description

### Case 1

**Figure 1:**
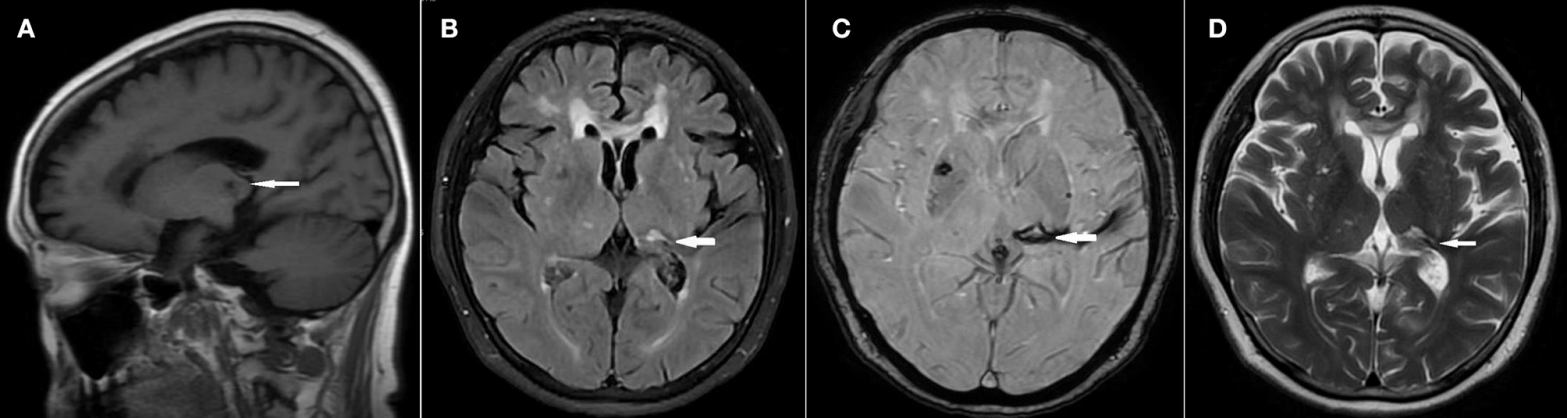
MRI without contrast showed ischemic stroke. The arrows indicated lesion of malacic at the left thalamus. **(A)** Saggital T1WI. **(B)** Axial T2 FLAIR. **(C)** 3D SWAN. **(D)** Axial T2WI.

In April 2020, a 57-year-old female patient initially experienced a sudden onset of a right-sided limb movement disorder and a lack of fluency in speech. The cephalometric CT scan at a local hospital showed thalamic hemorrhages. After conservative treatment, her speech function recovered, but she still felt poor physical strength and had an abnormal gait. She was then admitted to Beijing United Rehabilitation Hospital for further rehabilitation in March 2021. Two months after hospitalization, the patient developed moderate-to-severe pain [visual analog scale (VAS) 6–9] on the right side of her body, including the face, arm, trunk, and limbs, with no apparent cause. The pain was persistent and tightness-like, accompanied by paroxysmal pinprick-like pain occurring 30–50 times per day. The patient felt a painful wandering mass on the right plantar aspect of her foot and was unable to put weight on the right foot due to the pain. She also experienced paroxysmal knife-like pain (VAS 9) in the right upper arm, and moderate-to-severe tearing-like pain (VAS 6–9) in the right axilla and lateral chest wall. The magnetic resonance imaging (MRI) showed a malacic lesion in the left thalamus, and chronic ischemic lesions in the brainstem, bilateral basal ganglia, and periventricular white matter. Multiple white matter lesions in bilateral periventricular and subcortical white matter were observed, and Fazekas 3 was considered (Figure 1).

She was prescribed oral medications, including Gabapentin^Ⓡ^ (1,200 mg, three times a day), Duloxetine^Ⓡ^ (60 mg, once a day), Neurotropine^Ⓡ^ (8 u, twice a day), Tylenol^Ⓡ^ (1 tablet, as needed), and received traditional acupuncture, herbal medicine, and physical therapy. However, the pain was not alleviated. The patient frequently woke up at night due to pain outbreaks, which severely influenced her mood, sleep, and her ability to cooperate with rehabilitation treatment. Thus, she sought a consultation from the Division of Pain for further treatment.

During the consultation, the patient exhibited slower speech. Her right side of the body was hyperalgesic and could not tolerate cold when wiped with an alcohol swab. The muscle strength of the proximal right limb was grade 4, and the distal limb was grade 4^−^. The patient's right hand was unable to hold chopsticks or use a pen due to the contracture of the 3rd-5th fingers. Pressing pain was identified in the cervical spinous processes (C2–7), right transverse processes, and coracoid process of the right scapula. Similar symptoms were also detected in the right sternocleidomastoid, trapezius, and pectoralis major muscles. The abdomen was tense with pressing pain in the upper abdomen. The bilateral iliopsoas muscles, upper and lower back, and right hip experienced pressing pain. The patient also reported pressing pain in the posterior leg and on the right plantar.

**Figure 2:**
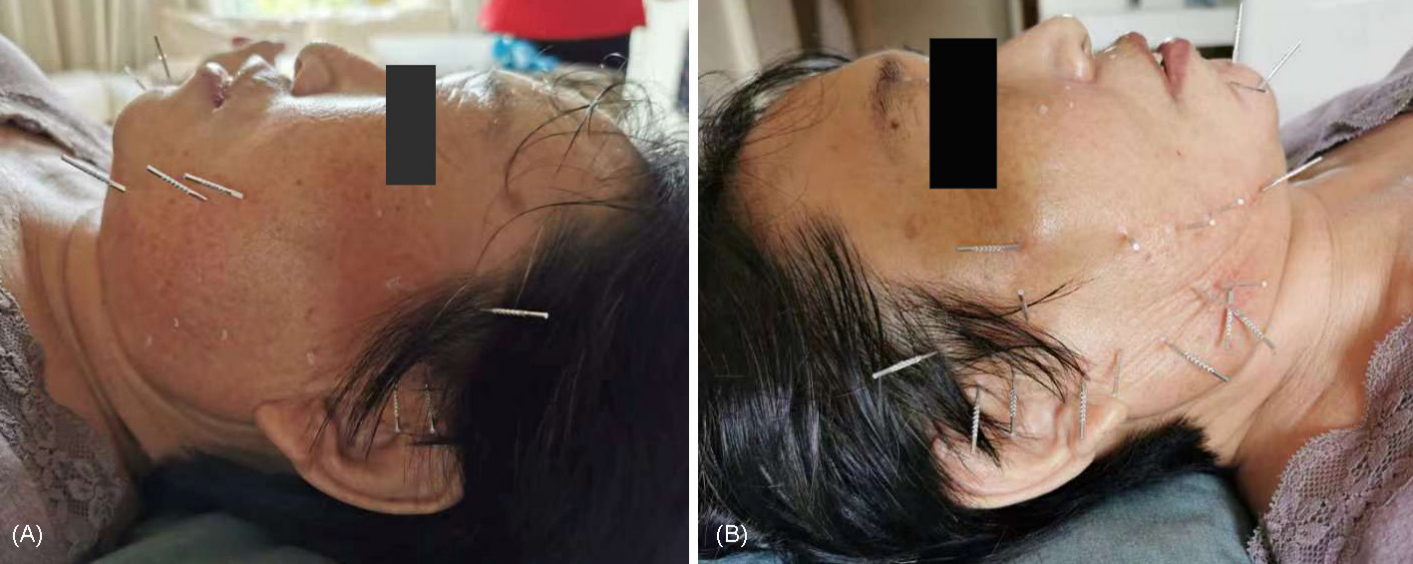
The selected acupoints for the first acupuncture treatment. **(A)** Acupoints were used on the cheek of left side. **(B)** Acupoints were selected on the right cheek.

After the first treatment with cheek acupuncture (Figure 2), the patient experienced significant relaxation on the right side of her body and a 60% reduction in spontaneous pain. The painful mass in the right foot shifted to the heel, decreasing in size and pain level. The patient could tolerate the pain in the right foot triggered by touching the ground. The first four sessions were administered once a week, followed by treatments every two weeks. The symptoms continuously improved during the treatments. Eight sessions later, the spontaneous pain in the right limb and trunk was reduced by 70–80%, and paroxysmal pinprick-like pain episodes occurred less than five times a day, lasting 2–3 seconds each time. There were no more nocturnal eruptive pain episodes. The patient only took Gabapentin 900 mg three times a day. The patient could stand only on right leg. The contracture of the right hand significantly improved, and the patient could perform certain fine activities, such as eating with chopsticks and writing. The walking gait was much better than before. Importantly, the patient's mood and sleep improved significantly, which facilitated rehabilitation training.

### Case 2

A 46-year-old female is a full-time mother and has been engaging in physical exercise for 2 h a day before sick, mainly to strengthen her abdominal, back, and gluteal muscles. She experienced on March 8, 2021 with severe pain in the right buttock, radiating to the area between the perineum and anus. The pain in the right gluteal region was moderate to severe, sharp pain (VAS 7–10). The radicular pain in the perineum was burning-like and electric shooting-like. Additionally, the patient experienced a slow onset of urination, anal swelling, and an increase in the frequency of stools (3–4 times a day). Turning over were difficult and lying down was in restricted positions due to the pain, which severely affected her sleep. She required regular walks or extra analgesic medicine at night to alleviate the pain and for sleep. An ultrasound of the superficial hip and a pelvic CT scan conducted in another hospital did not reveal any abnormalities. Blood sedimentation, C-reactive protein, and blood routine parameters were within the normal range. The patient had no significant medical history.

Over the last two weeks, she consulted several hospitals and took various analgesic medicines, including Imrecoxib^Ⓡ^, Aescuven^Ⓡ^, Mecobalamin^Ⓡ^, Tizanidine^Ⓡ^, TaiLeNing^Ⓡ^, and Pregabalin^Ⓡ^, which provided slight relief, but she still experienced moderate to severe pain (VAS 7–9).

The patient came for consultation (March 23, 2021) in our service with a limp. Physical examination revealed a positive Patrick's test, increased tone of the right rectus abdominis muscle, pressing pain on the right pubic tuberosity with evoked right perineal pain. Pressing pain was also detected in the right iliopsoas muscle, with radicular pain extending to the perineal area, as well as in the right lesser trochanter and right gluteal region (between the right sacrum and the right greater trochanter). Increased tone of the right erector spinae muscle and pain sensitization in the right perineal area were also observed. The pudendal neuralgia was considered.

Following cheek acupuncture, the burning-like pain in the right perineal area and the right hip pain disappeared, and the hip pressure pain was relieved by 90%. At the follow-up after treatment, the patient occasionally experienced mild electric shooting-like pain (VAS 2–3) in the right perineal area about 0–1 time per hour, along with mild deep hip pressure pain. On the fifth day after treatment, she discontinued the analgesic medications Imrecoxib^Ⓡ^, Aescuven^Ⓡ^, Mecobalamin^Ⓡ^, Tizanidine^Ⓡ^, and TaiLeNing^Ⓡ^, resulting in a slight increase in resting pain (VAS 2–3), hip pressure pain (VAS 6), and occasional mild electric shooting-like pain in the perineal area.

**Figure 3:**
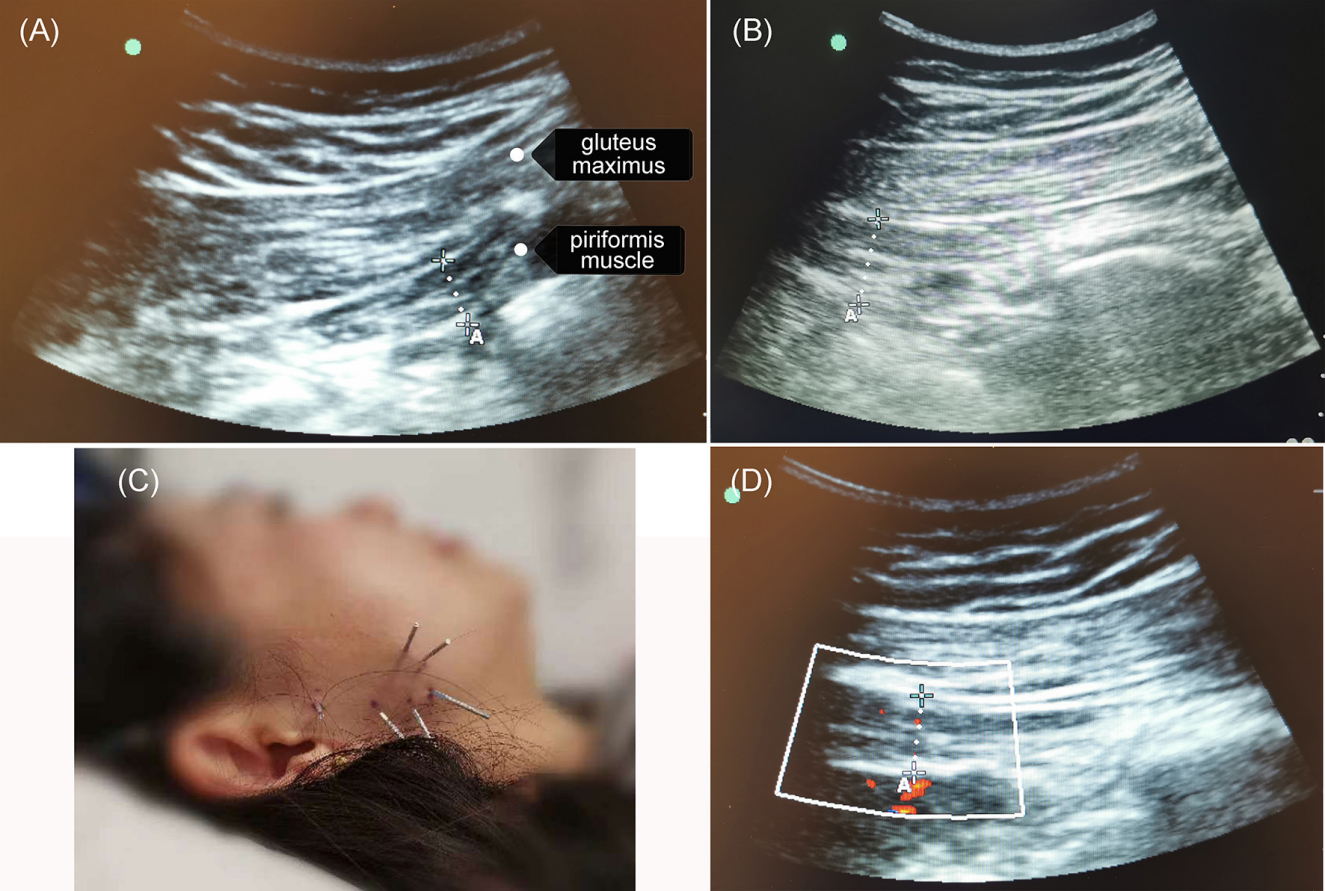
The cheek acupuncture reduced the thickness of the piriformis muscle. On day 7 after the first acupuncture session, an ultrasound was performed to measure the thickness of the piriformis muscle of the hip before and after the second session of treatment. **(A)** The piriformis muscle in the left hip (healthy) was 1.18 cm. **(B)** Before the second session of acupuncture, the thickness of the piriformis muscle in the right hip (injured) was 2.05 cm. **(C)** The selected acupoints for acupuncture treatment. **(D)** The piriformis muscle in the right hip (injured) decreased to 1.31 cm post-second treatment.

On the seventh day (March 30, 2021) after the initial acupuncture, an ultrasound was performed to measure the thickness of the piriformis muscle of the hip before the second session of acupuncture. The ultrasound probe was placed between the sacrum and the greater trochanter of the femur, where two layers of muscles, the gluteus maximus and piriformis muscles, were observed (Figure 3A). The piriformis muscle was identified by rotating the leg internally and externally. Color Doppler was used to determine the position of the inferior gluteal artery, next to which the thickness of the piriformis muscle was measured as 1.18 cm (left side, non-injured) and 2.05 cm (right side, injured) (Figures 3A, B), respectively. Then, cheek acupuncture treatment was performed for 30 mins (Figure 3C). After the treatment, the patient no longer experienced pain in the right perineal region and the right gluteus, and the gluteal pressure pain was relieved by 70%. Ultrasound showed the thickness of the piriformis muscle in right side decreased to 1.31 cm (Figure 3D). Follow-up on the third day after the second acupuncture session showed that the patient was pain-free and able to move without any positional limitations. Mild electric shooting-like pain occurred occasionally in the perineal area.

#### Acupuncture manipulation

The acupuncturist has a couple of years of experience in cheek acupuncture. Prior to performing acupuncture manipulation, a thorough physical examination of the patients was required to accurately localize the pain in the body. This helped in identifying the specific muscle(s) and soft tissues that were injured, and/or the nerve was compressed or influenced. The corresponding acupoint(s) on the cheek and the appropriate size of acupuncture needles were then selected for the acupuncture procedure. Once the needles were inserted, the analgesic effect was reevaluated through physical examination. If the pain did not fully or only partially alleviate, the depth and direction of needling needed to be adjusted, or additional needles closer to the acupoint were required until complete or significant pain relief was achieved. For each treatment, specific acupoints were selected based on the results of the physical examination. Generally, the needles were left in place for 30 mins during each session, and treatments were performed once every 1–2 weeks depending on the progress of the symptoms. Table 1 presents the standard acupoints of cheek acupuncture, their orientations, applications for various diseases, and acupoints that have been used (all sessions) for two patients in the present study.

**Table 1:** Standard acupoints of cheek acupuncture, their main applications and acupoints used in the present study for pain relief.

**Name**	**Orientation**	**Applications**	**Used for patients in this report**
Head point	1 inch above the upper edge of the middle point of the zygomatic arch	Headache, dizziness, toothache, insomnia, stress, anxiety, depression, stroke, etc.	P1
Upper energizer point	The cross of the posterior coronoid of the mandible and the lower edge of the zygomatic arch	Headache, cervical pain, chest pain, chest tightness, breast swelling and pain, tachycardia, arrhythmia, asthma, etc.	P1
Middle energizer point	The middle point of the connecting line between the upper and lower energizer acupoints	Stomach cramp, acute/chronic gastritis, heartburn with acidity,	P1
Lower energizer point	Anterior oblique line of the mandible	Abdominal bloating and pain, colitis, dysmenorrhea, pelvic inflammatory disease, menstrual irregularities, leukorrhea, gynecological disease	P1
Cervical point	Top edge of the root of the zygomatic arch	Neck pain, stiff neck after sleeping, cervical spondylosis, sore throat, dizziness, stress, scalene spasm, tinnitus, etc.	P1
Dorsal point	The cross of the lower edge of the zygomatic arch and the inferior capsule of the temporomandibular joint	Back pain, rhomboid muscle strain, chest tightness, shortness of breath, stomachache, heart palpitations, etc.	P1, P2
Lumbar point	The middle of the connecting line between dorsal and sacral points	Lower back pain, lumbar muscle strain, acute lumbar sprains, sciatica pain, herniated disc, etc	P1, P2
Sacral point	0.5 inch to the anterior & superior angle of the mandible	Sacrospinous muscle strain, lower back pain in women, injuries of sacroiliac ligament, bedwetting, prostatitis, etc.	P1, P2
Shoulder point	The middle point of the temporozygomatic sature	Shoulder pain, frozen shoulder, tendonitis of biceps brachii, synovitis of infra-acromion of scapula, tendonitis of supraspinatus muscle, etc.	P1
Elbow point	The middle point of the connecting line between the lateral canthus and the bottom of the zygomatic bone	Elbow pain, tennis elbow, golf elbow, wrist extensor tendonitis, wrist flexor tendonitis, etc.	
Wrist point	The point of nasolabial folds at the horizon level of the lower edge of nostrils	Wrist pain, injuries of wrist joint, carpal tunnel syndrome, finger pain	
Hand point	The middle of the connecting line between the middle point of the lower edge of nostril and vermilions border	Finger arthritis, tenosynovitis, finger numbness, hand numbness	
Hip point	1 inch of anterior & superior of the angle of the mandible on the masseteric tuberosity	Sciatica pain, wound-induced hip osteoarthritis, injury of piriform muscle, groin pain	P1, P2
Knee point	The middle point of the connecting line between the angle of the mandible and the chengjiang point	Knee pain, superficial fibular nerve pain, arthritis of knee joint, hamstring muscle injury, gastrocnemius muscle spasm, etc.	P1
Ankle point	1/3 proximity of the connecting line between the knee and Chengjiang points	Ankle joint sprain, ankle joint swelling and pain, ankle arthritis, Achilles tendinitis, heel pain	
Foot point	0.5 inch lateral to the Chengjiang point	Gout, metatarsals fascia sprain, plantar fasciitis, heel pain, toe pain	P1

P1, patient #1; P2, patient #2.

## Discussion

For neuropathic pain induced by damage to the CNS or peripheral nerve compression, the application of analgesic medicine or even decompression of the compressed nerve by surgery has been utilized. However, these approaches are either ineffective, have side effects, or can lead to subsequent complications. Specifically, in the present study, cheek acupuncture proved to be highly effective in alleviating severe pain resulting from both peripheral nerves compression and permanent damage/dysfunction to the CNS. In contrast, the analgesic medicines had limited effect, as described in the present study. Interestingly, cheek acupuncture provided immediate pain relief post-acupuncture and achieved clinical remission after several sessions. Additionally, cheek acupuncture is nearly painless compared to classical acupuncture, which often cause sensations of soreness, numbness, swelling, and pain at the site of needling. Thus, cheek acupuncture represents an efficient approach for relieving neuropathic pain, although substantial number of cases are still required to confirm its effect on neuralgia. However, the collective data from the cheek acupuncture team, widely distributed and practiced in China and abroad, have demonstrated its remarkable efficacy in relieving pain resulting not only from nerve disorders but also from other diseases (Wang et al., 2000; Ren et al., 2014; Wang, 2017; He and Li, 2018; Cai et al., 2020; Ding et al., 2020; Sun et al., 2022).

The cheek acupuncture technique was established by Dr. Yongzhou Wang. The core of this technique incorporates the Zang-fu viscera, the meridian theories, and the San Jiao (also known as the three energizers) theory of traditional Chinese medicine (TCM) (Wang, 2017). The author developed three major theoretical systems for cheek acupuncture: Da San Jiao, holography, and physical/mental integration. These systems are based on the physical-mental theory of Western medicine, the biological holographic theory developed by Zhang (1985), the anatomical structure of the human body, and the Qi pathway of TCM. Therefore, the indications for cheek acupuncture mainly focus on diseases occurring at these three levels.

In addition to its immediate pain-relieving effect and painless manipulation, the cheek acupuncture system utilizes only 16 acupoints (Wang, 2017), which is significantly fewer than the conventional acupuncture of TCM, which contains more than 300 acupoints. The orientation of these 16 acupoints on the buccal area is precisely determined according to the anatomical structure of the skull. Cheek acupuncture can be applied to relieve a wide range of pains induced by trauma, sports injuries, infection/inflammation, cancer, muscle injuries, post-operation recovery, and nerve compression/damage as described in this study (Wang, 2017). Furthermore, cheek acupuncture not only relieves local pain such as headaches and toothaches but also addresses pain in other parts of the body, including the neck, shoulders, extremities, back, lumbar area, and abdomen, regardless of whether the pain is acute or chronic, although the precise mechanism of action for pain relief by cheek acupuncture remains unclear.

Numerous studies have been conducted to investigate the mechanism through which acupuncture relieves pain [see review (Chen et al., 2020)], particularly in China in1960s and 1970s. The first national conference of acupuncture anesthesia research work held in Shanghai in February 1966 proposed that acupuncture could induce the release of potential molecules to relieve operative pain (Li, 2022). These molecules were later identified as endogenous opiate-like substances (OLS) and endorphin in 1970s by Chinese and American researchers, respectively. Han et al. further demonstrated that OLS in nucleus accumbens, amygdala, habenula, and periaqueductal gray (PAG) played a pivotal role in acupuncture analgesia (Han et al., 1980), and that cholecystokinin octapeptide (CCK8) acted as endogenous anti-opioid substance during acupuncture analgesia (Han et al., 1985). The interaction of endorphin with the opioid receptor, inhibiting the release of tachykinins (key proteins for pain transmission), is one widely accepted explanation for pain relief by acupuncture (Han, 2003; Sprouse-Blum et al., 2010). It has also been proposed that acupuncture-elicited endogenous opioids, serotonin, and norepinephrine released by immune cells at the local position and spinal level mediate persistent pain relief by desensitizing peripheral spinal nociceptor and by reducing the phosphorylation of the spinal N-methyl-D-aspartate receptor, respectively (Zhang et al., 2014; Lin et al., 2022). In line with this hypothesis, naloxone, an opioid antagonist, attenuated the analgesia effect of electro-acupuncture (Pomeranz and Chiu, 1976). Animal studies also have shown that adenosine, derived from rapid degradation of adenosine triphosphate (ATP) released by needle stimulation, may mediate the analgesic effect by binding to adenosine receptor (Tang et al., 2019). Other reports have also demonstrated that cheek acupuncture decreased the levels of 5-hydroxytryptamine (5-HT) and noradrenaline (NE) in patients with operative pain and in a rabbit model of rheumatoid arthritis (RA) respectively (Pu et al., 2018; Sun et al., 2022), indicating the potential involvement of both molecules in pain relief by acupuncture.

In addition to above neurochemical mechanism, the neurophysiological pathway is also considered to mediate pain relief by acupuncture. Similar to the transmission pathway of nociception and heat signals, it is known that the afferent signal from needling is transmitted to the spinal cord through the lateral spinothalamic tract, providing a structural basis for the potential interaction between afferent nociception and needling signals. In these models, the signal of needling stimulation is considered to be manipulated alongside the pain signal from the original injury in the body at different levels of the nervous system, including the spinal cord, brainstem, thalamus and cerebral cortex, respectively. Therefore, the afferent nociception to the CNS is down-regulated or the efferent pulse of inhibition from the CNS, activated by the afferent signals, is transmitted, resulting in pain relief. However, these mechanisms of action need to be further confirmed. Nevertheless, neither neurochemical nor neurophysiological mechanisms can fully explain the immediate and precise effect of cheek acupuncture on pain relief.

**Figure 4:**
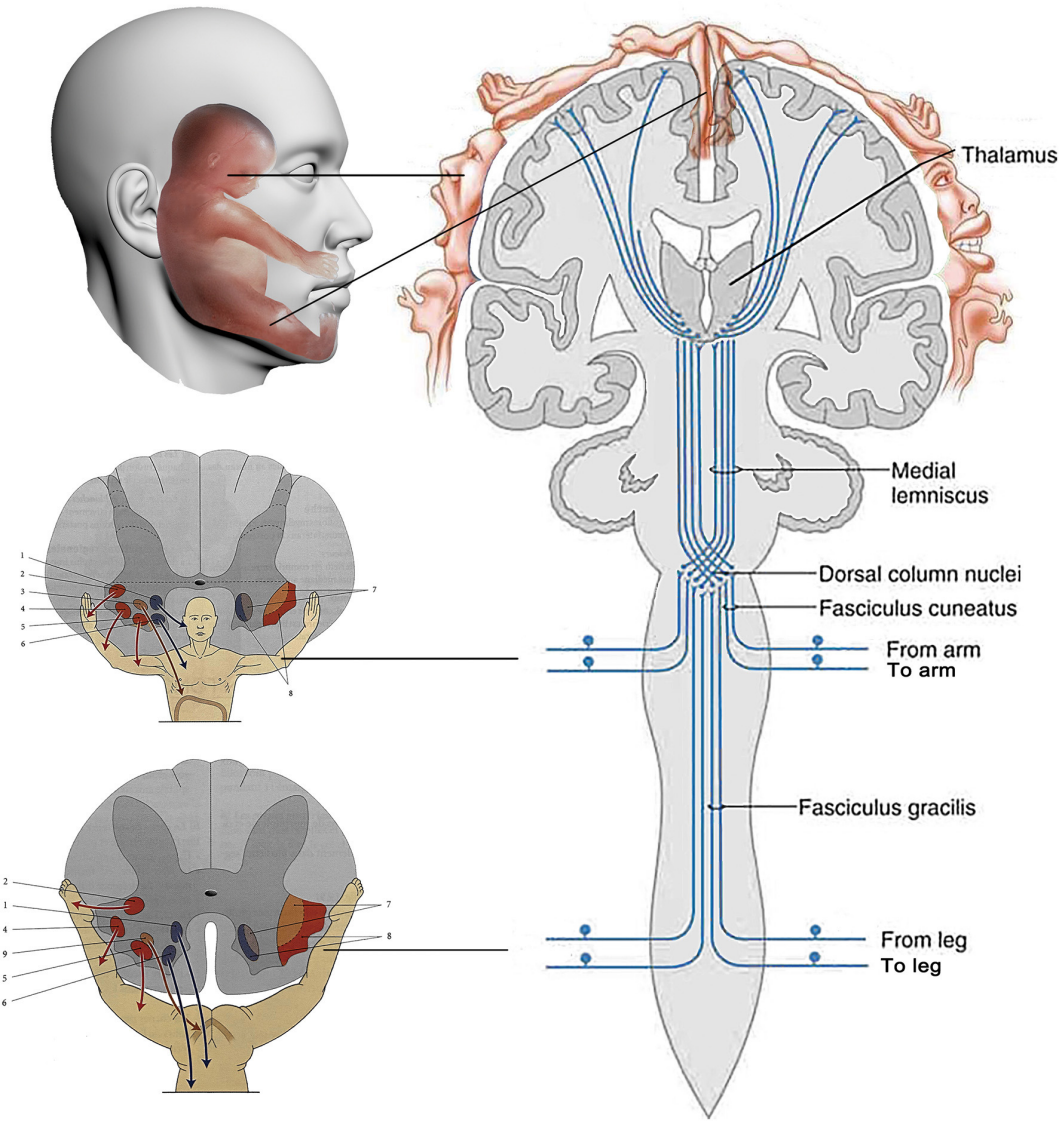
The simplified model of the mechanism of action of cheek acupuncture. The existence of biological holographic embryos (homunculi) as triplets at the levels of the local cheek, spinal cord, and cerebral cortex, which are the same as integrated body, is proposed. The simultaneous communication among three homunculi helps the integrated body sense pain signal and respond to needling, as well as transmit feedback and intervention signals, ultimately resulting in pain relief. The following are the labeled components of the model: 1. posterior-medial nucleus; 2. retro-posterolateral nucleus; 3. phrenic nuclei; 4. posterior-lateral nucleus; 5. anterolateral nucleus; 6. anteromedial core; 7. flexor muscle medulla; 8. extensor muscle medulla; 9. perineal core.

In the present study, we propose the biological holographic model to explain the immediate and precise analgesic effect of cheek acupuncture. In this model, we assume the existence of biological holographic embryos (homunculi) as triplets at the levels of the local cheek, spinal cord, and cerebral cortex. These homunculi have the same structure, organization, synchronized sensations and actions, as the integrated human body, which are mediated through afferent & efferent neurons. Nociception from local body injury is sequentially sensed, positioned, and analyzed by the homunculus localized at higher levels of the nervous system, including the cerebral cortex. Upon needle insertion, the buccal homunculus senses the needling stimulation via facial and trigeminal nerves distributed on the cheek. It immediately and directly “informs” homunculus in the cerebral cortex through afferent nerve fibers of the facial and trigeminal nerves, where the needling and nociception signals are communicated and analyzed. The feedback signal from the cerebral cortex is then transmitted directly to the homunculus in the buccal area via efferent fibers of the facial and trigeminal nerves. The intervention signal from cerebral cortex “informs” the homunculus in the spinal cord before precisely targeting the original injured site through efferent nerve fibers, resulting in analgesic, anti-inflammatory, tissue repair, and immune modulation effect. All these processes occur almost simultaneously among the homunculi and the integrated body. The simplified model is demonstrated in Figure 4. Our proposed model easily explains the immediate pain relief experienced by the integrated body once the needles are inserted at specific acupoint(s) of the homunculus on the cheek, corresponding to the localization of pain on the integrated body, regardless of whether the pain occurs locally or distantly in the body. Our proposed triplet homunculi model is supported by the evidence from: (1) the biological holographic embryo theory established by Prof. Yiqing Zhang in 1985 (Zhang, 1985); (2) the cloning of Dolly sheep using genomic DNA isolated from somatic cells, where the similar concept of biological holographic embryo was applied (Wilmut et al., 1997); (3) confirmation of homunculus on the cheeks and somatotopic homunculus in the cerebral cortex using precision functional magnetic resonance imaging (fMRI) methods (Wang, 2017; Gordon et al., 2023).

## Conclusion

This report introduces a new approach referred to as cheek acupuncture for relieving severe pain in two patients with either peripheral nerve compression or permanent damage/dysfunction to the CNS, when analgesic medicines were ineffective for both individuals. An immediate and accurate analgesia by cheek acupuncture was observed in these two cases, which is consistent with our collective data on pain relief by this technique (Wang et al., 2000; Ren et al., 2014; Wang, 2017; He and Li, 2018; Cai et al., 2020; Ding et al., 2020; Sun et al., 2022). Both patients achieved a clinical remission after 8 and 2 sessions of treatments, respectively. Complete recovery for case 2 and more improvement for case 1 may be achieved if more sessions of cheek acupuncture are provided.

Nevertheless, the features of standard acupoints, simplicity for manipulation, and accurate and immediate analgesia, make cheek acupuncture an efficient approach for relieving the pain suffered from disorders of the nervous system or other diseases. A biological holographic model of triplet homunculi is proposed to explain the mechanism of action of this technique.

## Data availability statement

The raw data supporting the conclusions of this article will be made available by the authors, without undue reservation.

## Ethics statement

Ethical approval was not required for the study involving human participants in accordance with local legislation. Written informed consent was obtained from individuals to participate in this study and for the publication of any potentially identifiable images or data included in this article.

## Author contributions

The cheek acupuncture was manipulated by LY. YWa and YWu designed the study. The manuscript was written by YWu.
